# Sigma factor M induces an ESX-4-focused regulon in *Mycobacterium abscessus*

**DOI:** 10.3389/ftubr.2026.1797268

**Published:** 2026-06-05

**Authors:** Jill G. Canestrari, Shawn Gianola, Keith M. Derbyshire, Todd A. Gray

**Affiliations:** 1 Division of Genetics, Wadsworth Center, New York State Department of Health, Albany, NY, United States; 2 Department of Biomedical Sciences, College of Integrated Health Sciences, University at Albany, Albany, NY, United States

**Keywords:** DUF3558, ESX-4, non-tuberculous mycobacteria, SigM, type VII secretion system

## Abstract

Mycobacteria encode as many as five distinct Type VII (ESX) secretion systems that function in nutrient acquisition, cell-cell interaction, membrane integrity and pathogenesis. The biological activities of ESX systems are determined by the effector proteins they secrete. ESX-4 is the ancestral secretion system and is conserved throughout mycobacteria, including *Mycobacterium abscessus* where it has been shown to support survival in phagosomes. ESX-4 and putative effector genes in *Mycobacterium tuberculosis* and *Mycobacterium smegmatis* are co-induced by a conserved sigma factor, SigM. Identifying the activities conferred by ESX-4 in promoting *M. abscessus* survival begins with identifying potential secreted substrates. We hypothesized that, in *M. abscessus*, SigM co-regulates the expression of genes encoding ESX-4 components and secreted effector proteins. We generated a precise deletion of the *sigM*-*rsmA* locus, encoding SigM and its dedicated anti-sigma factor. Performing RNA-seq using this deletion strain and a SigM-expressing complementation derivative, we identified SigM-responsive mRNAs in *M. abscessus*. Analysis of responsive promoters defined a consensus SigM-binding site, indicating direct induction by SigM. Together these data defined 15 SigM-targeted loci transcribing 50 genes with annotations and structures consistent with ESX secretion. While SigM induces multiple *esx4* locus promoters in *M. smegmatis*, only the promoter encoding the primary EsxU/EsxT secreted substrates are direct targets in *M. abscessus*, suggesting that production of the secretion apparatus is controlled by other transcription factors. The putative effector complexes identified here present strong candidates for the pathogenic roles previously reported for ESX-4 in *M. abscessus* infection models.

## Introduction

Type VII secretion systems, commonly known as ESX secretion systems, are complex assemblies of conserved proteins that span the cell membrane, translocating proteins out of the cytoplasm. ESX secretion systems are encoded by conserved genes clustered in loci that encode structurally similar core apparatus (1–5). Each *esx* locus also expresses a pair of small WXG100-proteins that are co-secreted by the core machinery (6, 7). In *Mycobacterium tuberculosis*, the WXG100-pair, EsxA and EsxB, are antigenic heterodimeric proteins secreted by ESX-1 (8–12). Slow-growing mycobacteria, such as *M. tuberculosis* and *M. marinum*, have five distinct ESX systems that have non-redundant roles in different processes (13, 14). ESX-1, ESX-4 and ESX-5 each contribute to pathogen-host interactions (8, 15) and ESX-3 functions in iron and zinc acquisition (16–18). The rapid-growing model organism, *M. smegmatis*, encodes three ESX systems (14). ESX-1 and ESX-4 are required for a unique conjugative process between donor and recipient strains, and ESX-3 acquires environmental iron and zinc (16, 19).

*M. abscessus* is a rapid-growing mycobacterial species and, similar to *M. smegmatis*, has evolved in environmental niches (20). However, individuals with certain chronic lung diseases are susceptible to infection by *M. abscessus* from aerosolized soil or water (21–24). In contrast to the obligate pathogen *M. tuberculosis*, *M. abscessus* is capable of both saprophytic and pathogenic existence. Further, *M. abscessus* encodes only two ESX secretion systems, ESX-3 and −4 (14, 25), raising the question of how fewer ESX secretion systems support the growth of *M. abscessus* in both the environment and as an intracellular pathogen. ESX-4 is regarded as the progenitor ESX system in mycobacteria that gave rise to paralogous secretion systems through successive duplication events followed by evolved specialization (10). It is possible that *M. abscessus* has retained an ancestral ESX composition with a subset of its secretion substrates conserved in other mycobacteria but secreted by descendant ESX systems.The importance of ESX-3 and ESX-4 in *M. abscessus* infection has been experimentally demonstrated in diverse models (25, 26). In a mouse model of *M. abscessus* infection, strains with mutations in *esx3* and *esx4* core genes were completely attenuated. Characterization of infected macrophages indicated that ESX-3 and ESX-4 blocked phagosome acidification and instead promoted phagosome lysis, allowing bacilli access to the cytosol (25). In support of this, *esx4* locus genes were identified in a screen of *M. abscessus* transposon insertion mutants for survival in phagosomes of macrophages or amoebae (27). While the ESX-4 secretion system clearly plays an important role in *M. abscessus* pathogenesis, the specific phagosomal survival activities provided by ESX-4 secretion have not been identified.

In other mycobacteria, an alternative sigma factor, SigM, co-induces ESX-4 secretion systems and putative secreted substrates. Microarray gene expression surveys of *M. tuberculosis* SigM identified the induction of *esxU* and *esxT*, which encode the WXG100 primary secretion substrate proteins for ESX-4, as well as other uncharacterized genes (28, 29). Clear functional roles for SigM and ESX-4 in *M. tuberculosis* remain elusive. In *M. smegmatis*, both SigM and ESX-4 were found to be essential in the recipient strain for conjugation (19). The SigM regulon in *M. smegmatis* includes six of seven *esx4* core locus genes, indicating that SigM regulates the synthesis of the ESX-4 apparatus in this species. In addition, 15 genes at nine loci in the genome were also induced by SigM and many encode proteins with structures and motifs consistent with ESX-secretion (30). In support of this, some of these non-*esx4* core genes were required for conjugation (30–33).

We hypothesized that in *M*. *abscessus*, SigM coordinates expression of genes that encode key components of the ESX-4 secretion system and those mediating phagosome disruption. To identify candidate genes that support survival in macrophages, we generated a *sigM-rsmA* deletion mutant in *M*. *abscessus* strain ATCC 19977 and a complement expressing *sigM* from a constitutive plasmid promoter. We identified differentially expressed loci and defined mRNAs regulated by SigM, each transcribed from a promoter with a consensus SigM-binding site, indicating direct regulation of the responsive genes. We further determined that proteins encoded in each locus have structural characteristics consistent with function as secretion substrates, annotated to have enzymatic activities that include hydrolase, kinase and toxin activities. Our data define a SigM regulon in *M. abscessus* that induces the primary ESX-4 secretion substrates EsxU and EsxT and co-induces putative secretion substrates that likely contribute to survival in infection.

## Methods

### Strains and plasmids

*Mycobacterium abscessus* ATCC 19977 strain (*Mab*) and its derivatives were grown in 7H9 medium supplemented with 10% OADC (Oleic acid, Albumin, Dextrose, Catalase) with 0.2% glycerol and 0.05% Tween-80 at 37 °C. The *sigM*-*rs* locus was deleted by a two-step process utilizing a suicide plasmid encoding resistance to zeocin, which contained segments flanking *sigM* from the *M. abscessus* genome (5050979-5051458 and 5053037-5053524) to promote recombination into the native locus. Recombinants were selected on 7H10 agar plates with 50 µg/mL zeocin and verified for the presence of the suicide vector by PCR amplification. Cultures of zeocin-resistant colonies were expanded through four serial passages in 7H9 broth without antibiotic selection to allow excisive recombination to occur. The plasmid also encoded *sacB* and passaged culture was spread on 7H10 agar plates supplemented with 5% sucrose. Zeocin-sensitive, sucrose-resistant colonies were identified by replica patching and were tested by PCR across the locus to verify the scarless deletion of 1580 bp spanning the *sigM*-*rsmA* genes. For complementation studies, *sigM* was expressed from the UV15/P_myc1_-tetO promoter on an L5 *attB* integrating plasmid encoding apramycin resistance (50 µg/mL) (34, 35). Colony morphology and size of the *sigM*-*rsmA* deletion or complementation strains were identical to the smooth ATCC 19977 WT *M. abscessus*.

### RNA-Seq

Replicate mid-log (O.D._600_ ∼0.7) cultures of WT, *ΔsigM-rsmA*, and complemented *M. abscessus* strains were grown at 37 °C with aeration and collected by centrifugation. Total RNA was extracted by bead-beating in TRIzol (Invitrogen). Residual genomic DNA was degraded by DNase digestion and tested for complete removal by PCR amplification. 500 ng of DNase-treated total RNA was depleted of rRNA using a mycospike probe mix with the NEBNext rRNA Depletion Kit for bacteria (NEB). Ribosomal RNA-depleted RNAs were processed for Illumina sequencing using the NEBNext Ultra II Kit (NEB). 10 million reads were obtained for each replicate and were trimmed (minimum read length of 40 nts and minimum Phred quality value of 30) by TrimGalore v.0.6.7 and mapped to the *M. abscessus* ATCC 19977 reference genome (CU458896.1). Gene expression was quantified by Salmon v.1.10.2 using the coding sequences for differential expression analyses by DESeq2. FastQ files used in this study are deposited in a NCBI Sequence Read Archive, BioProject accession number PRJNA1398377.

RNA-read depth plots were generated as previously described (33), with locus-specific adjustments made to accommodate varied locus lengths.

### SigM binding site identification

A FASTA file of 200 bp from the 5’ end of each induced operon was analyzed by MEME (https://m-suite.org/meme/tools/meme) (36). The default settings for the MEME search tool returns the top three enriched motifs. Only one sequence motif with a high probability score (4.1e^−079^) was returned and a single instance was identified in each promoter. The consensus was visualized using a DNA logo tool, aligned at the −35 GGAACCG motif, with 18 bp upstream and 33 bp downstream (https://weblogo.threeplusone.com/create.cgi) (37).

### Protein modeling

Predicted structures were provided by Uniprot or amino-acid sequence queries of Alphafold and Alphafold3 (38, 39). The models were visu, annotated and oriented using Chimera to produce images (40). A Chimera plug-in, ChopChop provided structural homology associations to supplement Uniprot homology annotations in Table 1 (41).

**Table 1:** The sigM regulon. SigM responsive genes (column 2) are organized by operon in genome order (column 1) and listing the Log_2_ fold change in expression upon SigM complementation of the deletion strain (column 3). The encoded protein for each gene is described by its Uniprot identifier (column 4), annotations derived from sequence or structural homologies (column 5) and the presence of WXG or YXXXD/E sequences (columns 6 and 7). Gene IDs were derived from the standard ATCC 19977 reference genome (CU458896.1) except *MAB_RS09705*, which was derived from reference genome (NC_010397.1). A complete list of direct SigM target operons and genes.

Operon	Gene ID	log2	Uniprot	Function/Family	WXG	YXXXD/E
**1**	Mab_0007	3.0	B1ME46	WXG100 family, EspB-like protein	Yes	Yes
	Mab_0008	3.8	B1ME47	Uncharacterized	No	No
	Mab_0009	2.6	B1ME48	DUF3558 domain protein, PknB-like	No	Yes
**2**	Mab_0010c	4.6	B1ME49	PPE family protein	Yes	No
	Mab_0011c	3.0	B1ME50	DUF3558 domain protein, PknH-like	No	Yes
	Mab_0012c	1.0	B1ME51	PE family protein	No	Yes
**3**	Mab_0147c	1.9	B1MEI3	ESX secretion protein EspG-like	Yes	No
	Mab_0148c	2.0	B1MEI4	PPE family protein	No	No
	Mab_0149c	1.0	B1MEI5	PE domain-containing protein	No	No
**4**	Mab_0251	0.6	B1MFF1	ESX-1 secretion EspB-EspK-like	Yes	No
	Mab_0252	2.0	B1MFF2	*α*/*β* hydrolase	Yes	Yes
	Mab_0253	2.1	B1MFF3	Lipoprotein LPPV	No	No
**5**	Mab_0375	3.2	B1MFS4	Uncharacterized	No	No
	Mab_0376	5.6	B1MFS5	WXG100 family type VII secretion target	Yes	Yes
**6**	Mab_1280c	3.5	B1MKS6	WXG100 family type VII secretion target	Yes	No
	Mab_1281c	2.9	B1MKS7	DUF3558 domain protein	No	No
	Mab_1283c	3.4	B1MKS9	WXG100 family type VII secretion target	No	Yes
**7**	Mab_1284c	2.3	B1MKT0	Lipoprotein-like	No	Yes
	Mab_1285c	3.0	B1MKT1	ESX-1 secretion EspA-like protein	Yes	Yes
	Mab_1286c	5.6	B1MKT2	WXG100 family protein	No	No
**8**	Mab_1828	4.6	B1MNL0	PE family protein	No	Yes
	Mab_1829	4.2	B1MNL1	DUF3558 domain protein, LipB-like	Yes	No
	Mab_1830	5.5	B1MNL2	PPE family protein	Yes	Yes
	Mab_1831	4.3	B1MNL3	WXG100 family protein	Yes	No
	Mab_1832	4.6	B1MNL4	NlpC/P60 domain-containing protein	Yes	No
	Mab_1833	4.3	B1MNL5	PknH-like protein	No	Yes
	Mab_1834	2.8	B1MNL6	Dps protein	No	Yes
**9**	Mab_1894c	2.4	B1MNS6	T6SS Tle1-like catalytic domain protein	No	No
	Mab_1895c	2.2	B1MNS7	PPE family protein	Yes	Yes
	Mab_RS09705	NA	A0AB74FHG3	DUF3558 domain protein, β lactamase	No	Yes
	Mab_1896c	1.2	B1MNS8	PE family protein-like	No	Yes
**10**	Mab_2771c	4.0	B1MC79	Chemotaxis Protein	No	Yes
	Mab_2772c	4.6	B1MC80	Uncharacterized	No	No
	Mab_2773c	4.4	B1MC81	YbaB/EbfC family EspL-like protein	No	No
**11**	Mab_3112	1.4	B1MD68	WXG100 family protein EsxF	Yes	No
	Mab_3113	1.6	B1MD69	WXG100 family protein EsxE	Yes	No
	Mab_3114	2.0	B1MD70	Bacterial toxin protein, CpnT homolog	No	No
	Mab_3115	1.9	B1MD71	Regulatory domain of aspartokinase	No	Yes
**12**	Mab_3663	3.0	B1MFZ2	ESX/PE protein-like	No	Yes
	Mab_3664	3.1	B1MFZ3	NlpC/P60 domain-containing protein	Yes	No
	Mab_3665	3.1	B1MFZ4	Uncharacterized, Rv3716c-like	No	No
	Mab_3666	3.4	B1MFZ5	YbaB/EbfC family EspL-like protein	No	No
**13**	Mab_3745c	2.2	B1MG74	Uncharacterized	No	No
	Mab_3746c	4.6	B1MG75	PE25/PPE41/EspG5-like	Yes	Yes
	Mab_3747c	2.8	B1MG76	ESX-1 secretion EsxB-like protein	No	Yes
**14**	Mab_3753c	4.7	B1MG82	WXG100 family protein EsxT	No	Yes
	Mab_3754c	4.7	B1MG83	WXG100 family protein EsxU	Yes	No
**15**	Mab_4829c	3.2	B1MMB2	PE/PPE Complex-like	No	Yes
	Mab_4830c	4.0	B1MMB3	WXG100 family protein	Yes	No
	Mab_4831c	3.9	B1MMB4	WXG100 family protein	Yes	No

## Results

**Figure 1:**
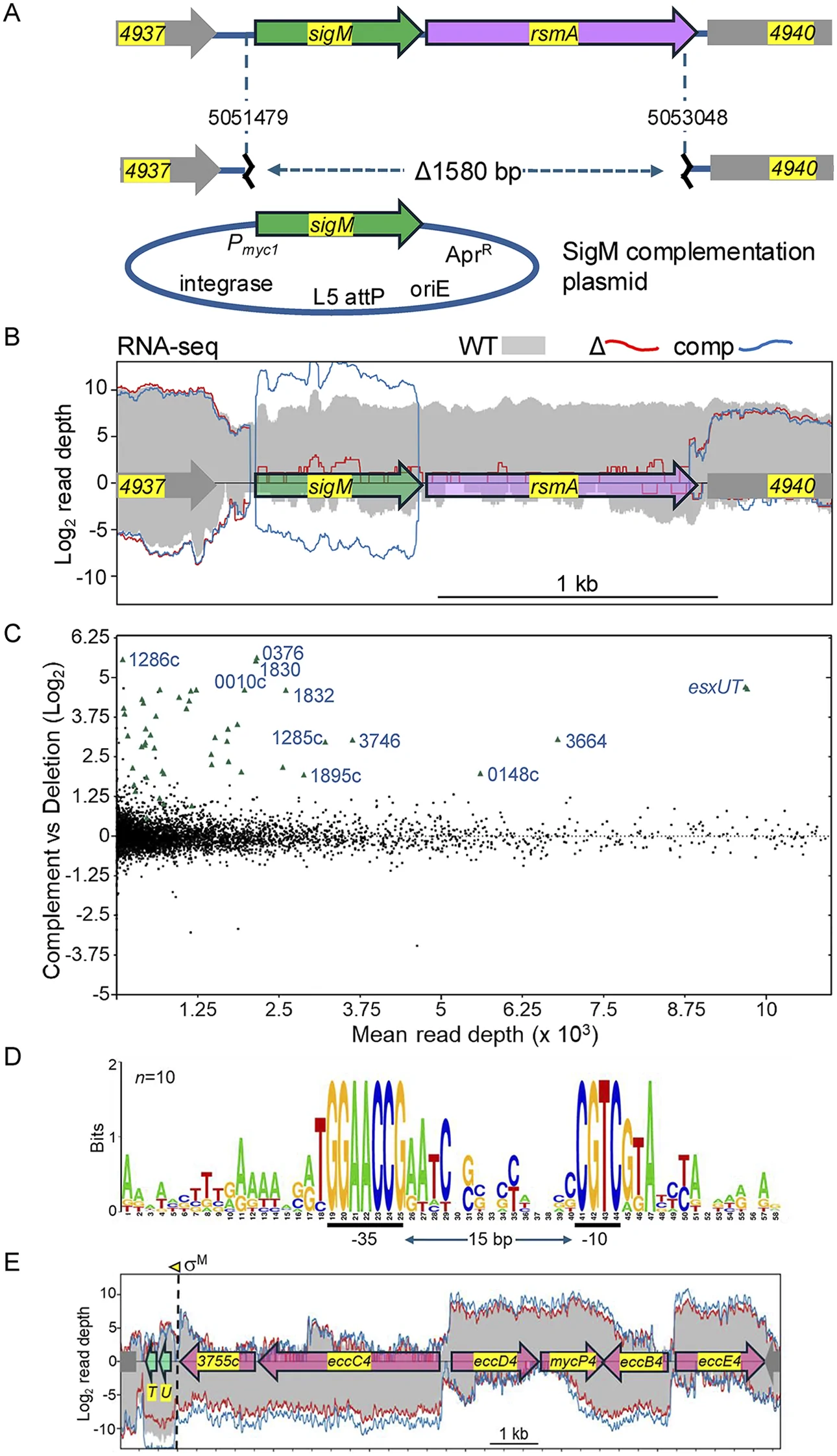
Transcriptional effects of sigM in the *M. abscessus esx4* locus. **(A)** Schematics of *sigM* constructs used in this study: the *sigM*-*rsmA* (Mab_4938-4939) locus, a precise deletion of the *sigM*-*rsmA* genes and an expression plasmid for *sigM* complementation. **(B)** Composite display of RNA-Seq profiles over the *sigM*-*rsmA* locus show the normalized expression from WT (gray shaded), deletion (red tracing) and complementation (blue tracing) derivatives. The negative strand reads over the *sigM* gene in the complementation sample may result from transcription arising from flanking plasmid sequences or the L5 *attB* insertion site. **(C)** An MA plot of differential gene expression of complementation vs deletion strain mRNAs. Direct SigM target genes are identified by green triangle symbols, annotated with Mab_ gene identification numbers in less congested areas. Not shown are 115 highly expressed, non-responsive genes (allowing a higher resolution plot of responsive genes) and the *sigM* gene (+ 11.9 Log_2_ and mean read depth 6550) as the independent variable. **(D)** A DNA logo of the consensus SigM binding site derived from ten responsive loci; remaining loci had single nucleotide variants. **(E)** Composite display of transcriptional effects of SigM over the *esx4* locus. One SigM binding site was identified in the *esxU*-*esxT* promoter (dashed line); the small triangle indicates direction of the −35/-10 elements. The genes directly induced by SigM are indicated by green-shaded arrows, with yellow highlight labels (U and T in this image placed below the very short gene arrows). The other six genes of the *esx4* locus are indicated by magenta arrows with yellow highlight gene labels. Flanking genes (dark gray) are shown for context. A 1 kb scale bar provides a locus size reference and normalized read depths are shown as Log_2_ values.

We generated a precise deletion of the *sigM-rsmA* locus (*ΔsigM*) of *M*. *abscessus* strain ATCC 19977 (Figure 1A). An integrating plasmid that expresses *sigM* from the *P*_myc1_ promoter was introduced into this deletion strain for complementation (+SigM). We cultured the wild-type (WT), the deletion strain and the deletion strain with the complementing plasmid to mid-log growth and processed the harvested RNA for RNA-seq analyses. Steady-state mRNA levels over the *sigM*-*rsmA* locus are shown in a consolidated display for WT (gray shading), *ΔsigM* (red tracing) and + SigM (blue tracing) strains (Figure 1B). We used DESeq2 to identify genes that were differentially expressed in deletion and complemented strains (Figure 1C and Table 1). Transcriptional profiles over loci identified by DESeq2 delineated responsive genes at each regulon locus. The promoter sequences of all SigM-responsive loci were analyzed for enriched sequence motifs, defining a consensus −35 and −10 SigM-binding site that matched the site reported for *M. smegmatis* (30) Ten promoters precisely matched the consensus (Figure 1D) and the other five had near-matches. We conclude that these 15 loci, totaling 50 genes, are direct transcriptional targets of SigM (Table 1).

In the *esx4* core gene locus, the *esxU-esxT* operon (*esxUT*) promoter includes a SigM binding site. The *esxUT* mRNA levels decrease in the *ΔsigM* samples relative to WT (Figure 1E, red tracing in lower left). The + SigM samples, conversely, expressed higher *esxUT* mRNA levels than WT (Figure 1E, blue tracing in lower left). Transcription through other genes in locus was not reduced in the *ΔsigM* samples but was slightly elevated in the + SigM samples (Figure 1E). This suggests that indirect effects of SigM expression may marginally enhance basal transcription of these *esx4* mRNAs.

**Figure 2:**
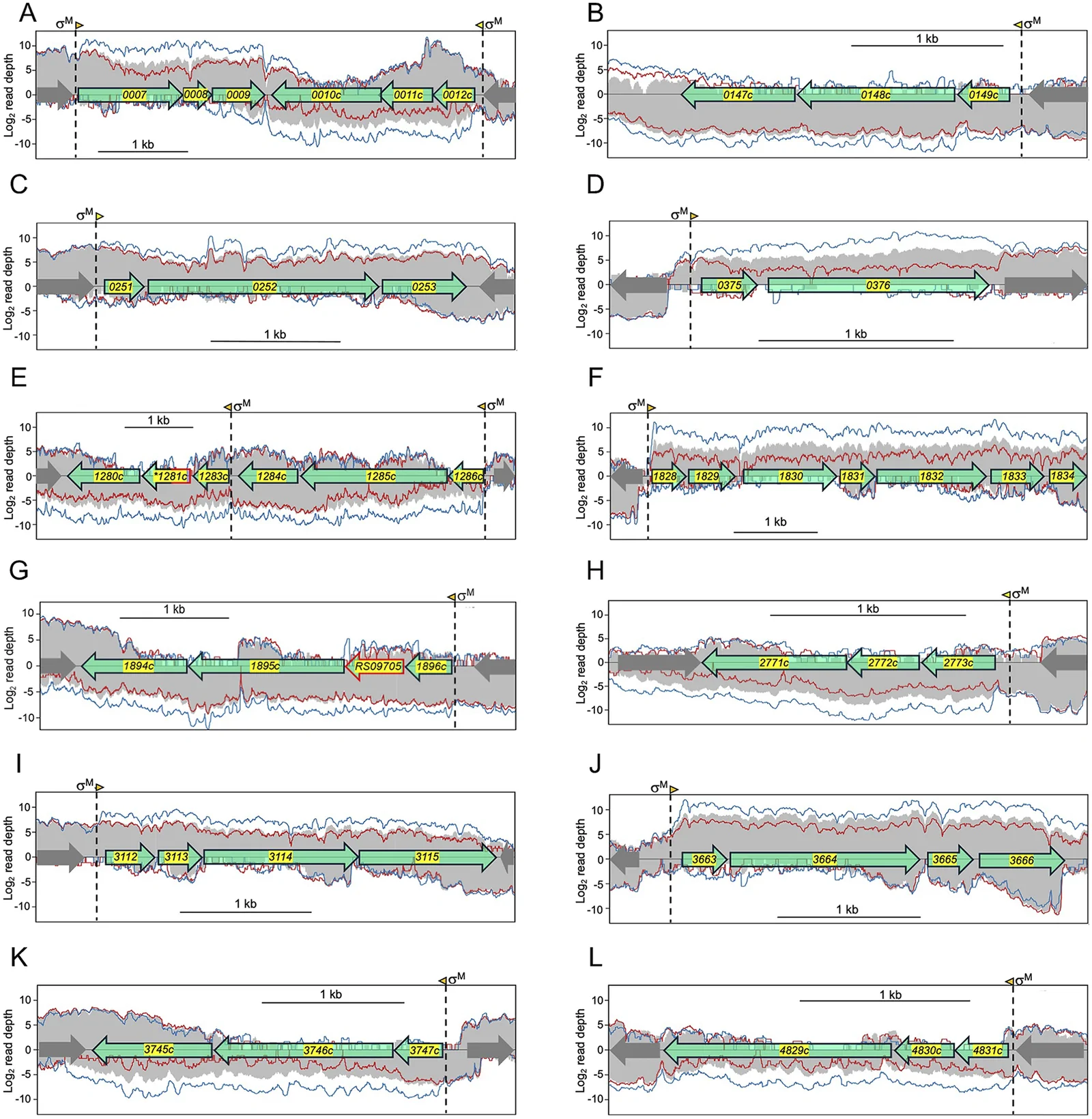
Composites displays of individual sigM-targeted putative effector loci. **(A)** *Mab_0007 – 0012c* show two three-gene convergent operons. *Mab_0007-0009* has a consensus SigM binding site (orange triangle above the dashed line) and *Mab_0012c – 0010c* has a near-consensus match (yellow triangle above the dashed line). **(B)** *Mab_0149c – 0147c*. **(C)** *Mab_0251–0253*. **(D)** *Mab_0375–0376*. **(E)** *Mab_1286c – 1280c* indicates two three-gene negative-strand operons. The red outline of *Mab_1281c* indicates a corrected N-terminal extension of the CU458896.1 annotation to match the annotation in NC_010397.1 as *MAB_RS06640*. **(F)** *Mab_1828–1834*. **(G)** *Mab_1896c – 1894c*. The gene represented by RS090705 was not predicted in CU458896.1 but was identified in NC_010397.1. **(H)** *Mab_2773c – 2771c.* **(I)** *Mab_3112–3115*. **(J)** *Mab_3663–3666.* **(K)** *Mab_3747c – 3745c*. **(L)** *Mab_4831c – 4829c*.

The transcriptional response at each of the remaining 14 loci is shown in consolidated profile panels in Figure 2. In each instance, complementation by SigM increased transcript levels relative to the WT (the blue tracing exceeding the gray shading) samples through the operon, beginning immediately adjacent to the identified SigM-binding site (dashed vertical line in each panel). These profiles individually and collectively demonstrate that SigM-binding to its cognate −35/-10 binding site in promoters induces transcription of the operon. As in the *esx4* locus, deletion of *sigM* only slightly decreased expression of some operon mRNAs below that of WT (e.g., Figures 2D, F – indicating partial SigM dependence), while other loci were unaffected even though they are transcribed from *sigM* promoters (e.g., Figures 2B, G). A notable feature in the GC-rich mycobacterial genome is the observation of an A-rich region 5’ of the −35 site in the SigM binding site sequence logo (Figure 1D), which we speculate may play a role in regulon gene expression.

**Figure 3:**
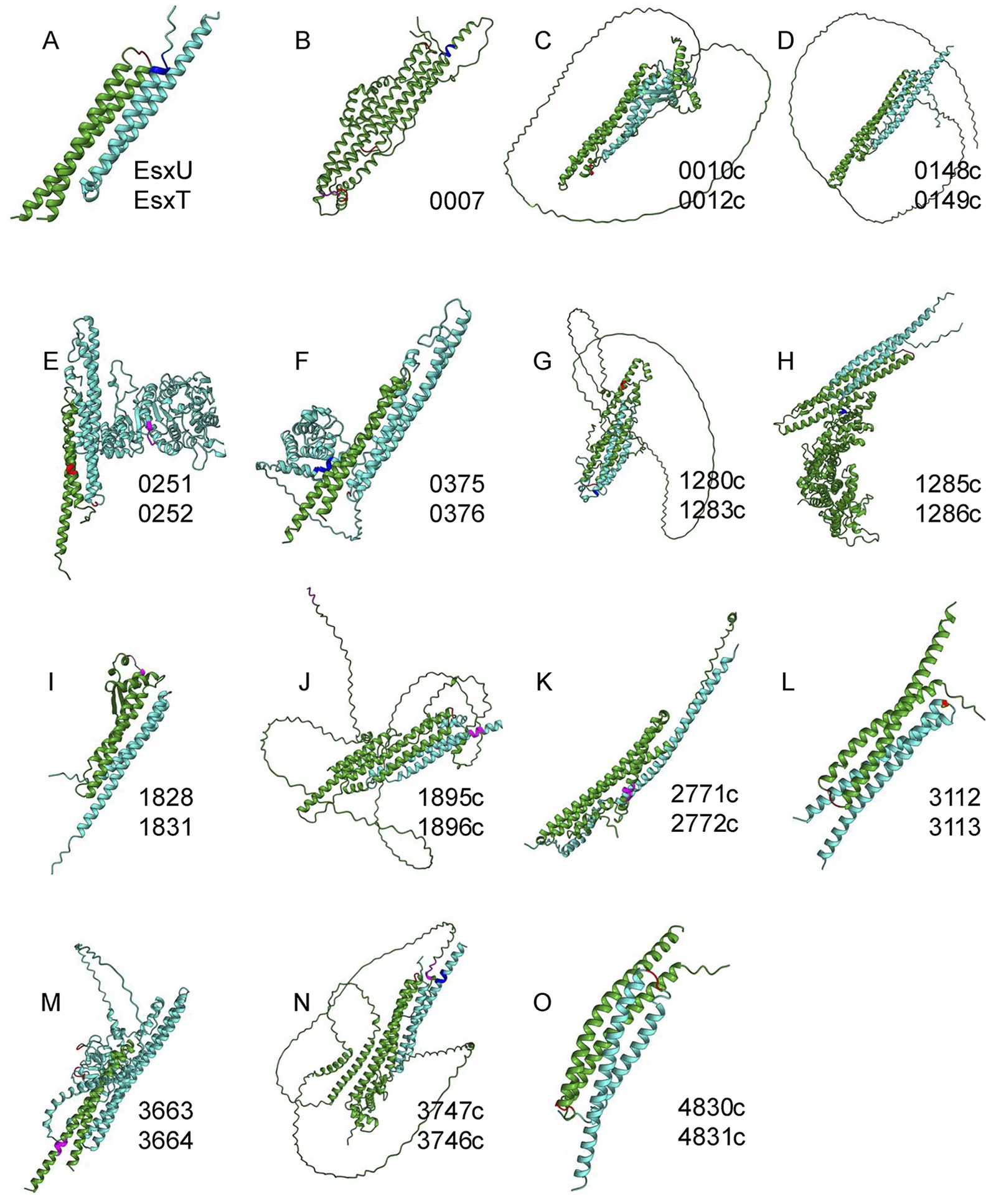
Structural predictions for sigM-regulon target proteins and complexes consistent with ESX secretion. Alphafold and Alphafold 3 were used to predict the protein conformations and interactions, respectively. Possible ESX secretion sequences are indicated where present (WXG in red, YXXXD in magenta and YXXXE in blue). The protein encoded by the gene with the lower number is shown in green to allow it to be distinguished from the binding partner colored with cyan. Each structure was independently sized and oriented. **(A)** Mab_3754c and 3753c (EsxU and EsxT) represent the standard four-helix bundle and WXG and YXXXE sequences. **(B)** Mab_0007 is predicted to be a monomeric secretion substrate. **(C)** Mab_0010c and Mab_0012c, with an unstructured region from Mab_0010c depicted as a loop around the helix bundle. **(D)** Mab_0148c and Mab_0149c. **(E)** Mab_0251 and Mab_0252, which also encodes an *α*/*β* hydrolase domain projecting from the helix bundle. **(F)** Mab_0375 and Mab_0376. **(G)** Mab_1280c and Mab_1283c. **(H)** Mab_1285c and Mab_1286c. **(I)** Mab_1828 and Mab_1831. **(J)** Mab_1895c and Mab_1896c. **(K)** Mab_2771c and Mab 2772c. **(L)** Mab_3112 and Mab_3113 (EsxF and EsxE). **(M)** Mab_3663 and Mab_3664. **(N)** Mab_3746c and Mab_3747c. **(O)** Mab_4830c and Mab_4831c.

ESX secretion substrates described in other mycobacteria feature a four-helix bundle structure and a bipartite secretion signal sequence comprising tryptophan-X-glycine (WXG) and tyrosine-XXX-aspartic/glutamic acid (YXXXD/E) motifs (53). Substrates are divided into three primary classes (42–44). Esx proteins belong to the WXG100 class and are expressed as tandem genes in individual *esx* loci and elsewhere in the genome. A predicted (Alphafold 3) image of EsxU and EsxT, of the WXG100 heterodimer family (6, 7), illustrates the hallmarks of the four-helix bundle with juxtaposed WXG and YXXXD/E sequences (Figure 3A). Other predicted Esx proteins are modeled in Figure 3 (I, K, L and O). Mab_3112 and Mab_3113 (Figure 3L) are homologs of *M. tuberculosis* EsxF and EsxE, respectively.

The PE/PPE (so named for their proline and glutamic acid-rich N-termini) class of proteins include a ∼100 amino acid PE protein that dimerizes with a larger PPE partner to produce a four-helix bundle and secretion signal. PE/PPE proteins are a heterogenous family that can be further subdivided by size and function and have been described in ternary complexes with cognate EspG chaperones (45, 46). Most of the *M. abscessus* SigM regulon proteins are predicted to have PE/PPE-like structures. Mab_0010c is annotated as a PPE family protein and is shown paired with Mab_0012c, a predicted PE protein (Figure 3C). The modeled Mab_0012c and Mab_0010c structure is the first of several PE/PPE heterodimer predictions that have a large unstructured domain. Other predicted PE/PPE-class secretion substrates encoded in the SigM regulon are shown in Figure 3 (D, E, F, G, H, J, M and N). The Mab_0149c and Mab_0148c PE/PPE proteins are co-expressed with a predicted EspG-like chaperone encoded by Mab_0147c suggesting association as a ternary complex (45, 46).

A monomeric class of ESX secretion substrate encodes the four-helix bundle and secretion signal in a single protein. EspB represents this class and has been reported to interact with EspK, each of which are encoded in *esx1* loci in other mycobacterial species (47, 48). Mab_0007 expresses an EspB-like protein (Table 1) (Figure 3B).

## Discussion

We identified potential ESX-secretion substrates of *M. abscessus* by defining the SigM regulon as 15 loci encoding 50 genes, producing proteins or protein complexes predicted to function as ESX secretion substrates. Thus, as has been shown for *M. smegmatis* and *M. tuberculosis*, SigM also coordinates the induction of an ESX response in *M. abscessus*.

Our data also support annotation updates for two SigM-regulated genes. In one locus, the ATCC 19977 reference genome (CU458896.1) predicts that a positive-strand gene, Mab_1282, separates the negative strand genes, Mab_1283c and Mab_1281c. An alternative reference genome, (NC_010397.1), correctly identifies a gene it calls MAB_RS06640, that extends Mab_1281c to include 108 N-terminal amino acids. Our mRNA profile over this region shows continuous transcription consistent with MAB_RS06640 (Figure 2E, red outline arrow reflects the N-terminal extension). Thus, we conclude that the predicted Mab_1282 is likely incorrect. The reference genome, NC_010397.1, also correctly identifies a 534-bp gene that is co-transcribed with Mab_1896c – 1894c. This gene encodes a 178 amino acid protein, MAB_RS09705, annotated as a DUF3558 protein (Figure 2G and Table 1).

Each SigM-regulon locus encodes proteins consistent with ESX secretion. While it is premature to assign functions, there are recurrent patterns that suggest two structural themes. The first is the six instances of DUF3558 domain-containing proteins (Table 1). There are 11 loci encoding proteins with DUF3558 annotations in *M. abscessus* (NC_010397.1), showing that this domain is highly enriched in the SigM regulon. The high frequency of DUF3558 proteins that are co-expressed with ESX secretion substrates strongly suggests a fundamental functional link. Determining whether DUF3558 functions as a secretion chaperone, or as an effector protein will require directed study. DUF3558 domains range from 177 to 195 amino acids and are found throughout Actinomycetes, including *Nocardia* spp. and *Gordonia* spp.

A second emerging theme is that some genes encode catabolic activities that we speculate could facilitate envelope remodeling of the host bacterium or, conversely, damage neighboring cells (Table 1, e.g., those encoded in operons 4, 8, 9, 11, and 12). For example, operons 8 and 12 encode predicted NlpC/P60 peptidases that may function in cell wall hydrolysis and remodeling (49). A SigM-regulon NlpC/P60 protein in *M. smegmatis* is induced in the recipient upon donor contact and is required for conjugation (30). Another example is operon 11, which has a homologous operon in *M. tuberculosis* that co-transcribes the *esxF*-*esxE-cpnT*-*ift* genes encoding an ESX-secreted necrosis-inducing toxin that is required for survival in macrophages. We suggest that in *M. abscessus*, Mab_3114 toxin secretion is co-dependent on the Esx proteins encoded by Mab_3112 and Mab_3113. Operon 4 also encodes a predicted hydrolase. *α*/*β* hydrolase folds are found in glycopeptide lysin proteins expressed by mycobacteriophages (50) and have been previously associated with PE/PPE proteins (51), consistent with roles in mycobacterial cell wall hydrolysis. Twelve of the 15 operons are present in 90 or more of a 96 *M. abscessus* cohort that served to provide a pangenome survey of the species, indicating that most are conserved and that our findings from ATCC19977 have broad relevance (52). Despite their conservation in distant mycobacteria, operons 8 (NlpC/P60) and 11 (CpnT), are present in only 39 and 72 isolates, respectively. Specific assays will be needed to determine the biochemical activities and biological roles of these proteins in the environment and in infection.

Several observations from this study support the evolutionary model of ESX-4 as a progenitor secretion system for specialized, spinoff ESX systems formed by subsequent *esx* locus duplication and divergence. Basal monoculture expression of *esx4* genes in *M. smegmatis* is nearly undetectable until donor contact induces the recipient SigM regulon, which includes multiple mRNAs within the *esx4* core locus (30). By contrast, in *M. abscessus*, SigM directly induces only *esxUT* above the marked basal transcription of genes in the *esx4* locus. While each regulon operon we identified is directly induced by SigM, none are entirely dependent on SigM, leading us to speculate that a partially redundant sigma factor also activates SigM promoters. Collectively, these observations indicate that *M. smegmatis* requires ESX-4 function specifically in conditions that activate SigM, whereas *M. abscessus* has additional roles for ESX-4 secretion in situations separate from those that activate SigM. Further, we note that most *M. abscessus* SigM operons encode PE/PPE class substrates, which are predominantly secreted by ESX-5 secretion systems (2, 45, 46). Other loci encode EspB and EspG homologs, which are associated with ESX-1 and ESX-5 in other mycobacteria (45–48). This is consistent with *M. abscessus* representing an ancestral ESX-4 system that performs a broad range of functions that have subsequently evolved to be accomplished by derivative paralogous systems in other mycobacteria.

The conservation of this extended regulon in *M. abscessus* suggests that it is key for bacterial survival in its natural environment and during opportunistic infection. A basal level of regulon transcription provides steady expression suitable for rapid stimulus-responses, while specific contacts and/or environmental conditions activate SigM in *M. abscessus* to boost expression of ESX-4 secreted proteins. The identification of these SigM-regulon proteins is a significant step toward understanding these interactions and, potentially, to therapeutically disrupting them.

## Data Availability

The datasets presented in this study can be found in online repositories. The names of the repository/repositories and accession number(s) can be found in the article/Supplementary Material.
